# The Role of Amino Acids in the Diagnosis, Risk Assessment, and Treatment of Breast Cancer: A Review

**DOI:** 10.3390/cimb45090474

**Published:** 2023-09-13

**Authors:** Lyudmila V. Bel’skaya, Ivan A. Gundyrev, Denis V. Solomatin

**Affiliations:** 1 Biochemistry Research Laboratory, Omsk State Pedagogical University, 644099 Omsk, Russia; ivangundyrev@yandex.ru; 2 Department of Mathematics and Mathematics Teaching Methods, Omsk State Pedagogical University, 644043 Omsk, Russia; denis_2001j@bk.ru

**Keywords:** amino acids, breast cancer, diagnosis, imaging, risk, prognosis, treatment

## Abstract

This review summarizes the role of amino acids in the diagnosis, risk assessment, imaging, and treatment of breast cancer. It was shown that the content of individual amino acids changes in breast cancer by an average of 10–15% compared with healthy controls. For some amino acids (Thr, Arg, Met, and Ser), an increase in concentration is more often observed in breast cancer, and for others, a decrease is observed (Asp, Pro, Trp, and His). The accuracy of diagnostics using individual amino acids is low and increases when a number of amino acids are combined with each other or with other metabolites. Gln/Glu, Asp, Arg, Leu/Ile, Lys, and Orn have the greatest significance in assessing the risk of breast cancer. The variability in the amino acid composition of biological fluids was shown to depend on the breast cancer phenotype, as well as the age, race, and menopausal status of patients. In general, the analysis of changes in the amino acid metabolism in breast cancer is a promising strategy not only for diagnosis, but also for developing new therapeutic agents, monitoring the treatment process, correcting complications after treatment, and evaluating survival rates.

## 1. Introduction

Breast cancer (BC) remains the leading malignant neoplasm in women and the second leading cause of death from cancer among women worldwide [1,2]. Treatment for breast cancer has improved in recent years, but its high mortality remains a concern. Existing treatments for breast cancer often lead to intractable drug resistance. Therefore, the need for methods of early diagnosis and the identification of risk factors and groups are still relevant tasks. To solve these problems, scientists began to study the metabolic pathway in breast cancer. The connection between breast cancer and metabolic pathways may also lead to the discovery of new therapeutic possibilities and targets [3,4].

Recently, much attention has been paid to metabolomics as an effective tool for differentiating samples from patients with breast cancer from normal samples [5,6,7,8,9,10,11]. For example, a statistically significant difference in 24 metabolites in breast cancer compared with the control group was found by Dougan et al. [12], and a statistically significant difference in 78 metabolites was confirmed by Shen et al. [8]. Interestingly, the levels of metabolites are unique for different types of cancer, which could be a new way to classify tumors [13]. Jobard et al. identified nine metabolites to differentiate between metastatic and early breast cancer, although a separation of breast cancer phenotypes based on metabolomics is still controversial [14,15]. Several metabolomic studies of plasma/serum breast cancer have been performed, mainly aimed at distinguishing between the subtypes of breast cancer [16], metastatic breast cancer [8,14,17,18,19,20], recurrence [21,22], response to neoadjuvant chemotherapy [23,24], etc. An important part of the metabolomic profile, which makes it possible to discriminate between BC patients and healthy controls, is represented by amino acids. Amino acids in breast cancer are determined in the tissue [25,26,27], serum [7,28,29,30], plasma [6,31,32,33,34], saliva [35,36,37,38], and urine [39,40,41]. Amino acids not only play a vital role in the synthesis of biological molecules such as proteins in malignant cancer cells, but they are also important metabolites for immune cell activation and antitumor activity in the tumor microenvironment. Abnormal changes in the amino acid metabolism are closely associated with the onset and development of tumors and immunity [42]. An amino acid can supply sources of nitrogen and carbon for biosynthesis or satisfy the energy requirement for the rapid growth of tumor cells [43].

In this review, we focus on the role of amino acids in the diagnosis, risk assessment, imaging, and treatment of breast cancer.

## 2. Diagnostic Value of Determination of Amino Acids in Biological Fluids

### 2.1. Amino Acid Composition of Serum and Blood Plasma in Breast Cancer

The determination of the content of amino acids in serum and blood plasma was carried out in a large number of studies (Table 1).

For example, Kubota et al. showed that in breast cancer, the total content of amino acids (TAA) in plasma increases (3035 ± 118 vs. 2529 ± 115 nmol/mL), as do the contents of all major groups of amino acids: essential amino acids (EAAs), branched-chain amino acids (BCAAs), aromatic amino acids (AAAs), and gluconeogenic amino acids (GAAs) [44]. Similarly, a number of studies have also noted an increase in the amino acid content compared to healthy controls [50,61]. According to other data, in patients with breast cancer, a decrease in the plasma levels of branched-chain amino acids (BCAAs) was observed [57]. Lai et al. [62] showed that a decrease in the content of seven amino acids (Ala, His, Thr, Arg, Pro, Glu, and Gly) occurs more than six times more often than their increase. In this regard, it is noteworthy that these seven amino acids play more important roles than others during changes in the protein metabolism in cancer patients. In a review by Yang et al., an analysis of the most common biomarkers of breast cancer was carried out [63]. Based on clinical metabolic studies, Tyr and Ala shared the highest frequency, indicating that they may be sensitive metabolites in the diagnosis of breast cancer. Other amino acids are mentioned less frequently, and the frequency of occurrence decreases in the following order: Tyr (6/6), Ala (6/5), Glu (6/4), Val (4/6), Phe (5/4), Gln (4/5), Lys (3/6), Ile (4/4), His (3/4), Gly (4/2), Arg (3/3), Asn (4/2), Pro (3/3), Ser (5/1), Leu (2/4), Trp (1/5), Thr (2/3), Asp (3/1), Orn (2/2), and Cys (2/2). It should be noted that in different studies, the level of amino acids changes in different directions: the first digit corresponds to up-regulation, and the second digit corresponds to down-regulation [63]. Thus, the existing data from the literature provide conflicting information about the nature of changes in the content of amino acids in breast cancer.

In different studies, both the contents of individual amino acids and the complete amino acid profile of plasma and/or serum were evaluated. It has been shown, for example, that the arginine levels were significantly lower and the ornithine levels were significantly higher in breast cancer patients than in control patients [64]. Differences between breast cancer cases and controls were observed for the levels of Trp, Thr, Ala, Gly, Pro, Ile, Leu, and Val [34]. Miyagi et al. observed significant changes in the amino acid profiles between cancer patients and controls. They found that the concentrations of Thr, Pro, Ser, Gly, Ala, and Orn were significantly higher, but the concentrations of Gln, Trp, His, Phe, and Tyr were significantly lower compared to the control. Compared with the data from Lai et al., they found similarities in decreasing His and Gln levels and increasing Pro and Ala levels in breast cancer patients. Budczies et al. demonstrated that changes in the amino acid metabolism were associated with at least a 1.9-fold increase in 16 amino acids in breast cancer compared with healthy breast tissue [50,65]. 

The summarized results on the amino acid composition of serum/plasma in breast cancer compared with healthy controls are shown in Table 1.

**Figure 1:**
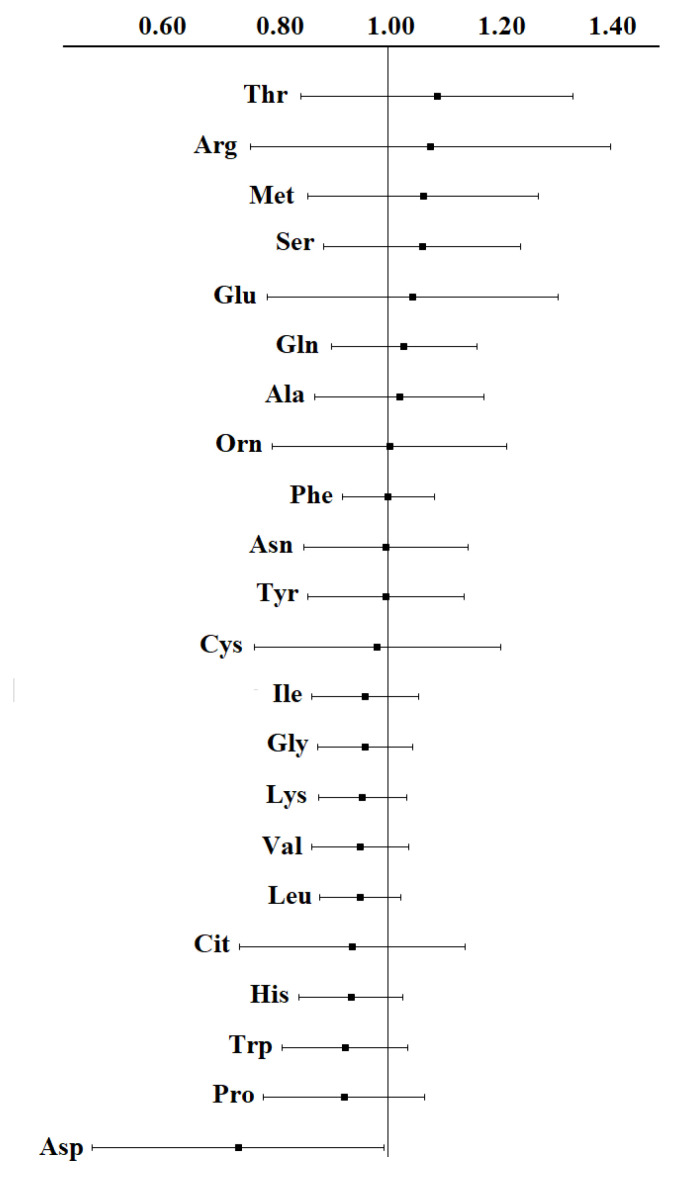
Fold change BC/control (data were averaged over [7,8,28,33,34,44,46,47,48,49,51,52,53,56,57,60]).

To compare the change in the contents of individual amino acids in breast cancer according to different authors, we compared the relative contents of amino acids in relation to healthy controls (Table 2). It can be seen that a significant change in the concentration is rarely observed, for example, an increase in the contents of Arg [52,56], Thr [52], and Ser [33] by 2 times, a decrease in the content of Asp by 1.5 times [46,56], etc. In most cases, the change in concentration is up to 10–15% compared to the control. We calculated the average change in concentration and the interval of variation for each amino acid and plotted them on a diagram (Figure 1). Thus, it is possible to assess the nature of changes in the concentrations of individual amino acids in breast cancer.

It can be seen that for some amino acids (Thr, Arg, Met, and Ser), an increase in concentration is more often observed in breast cancer compared with a healthy control, and for other amino acids, a decrease is observed (Asp, Pro, Trp, and His) (Figure 1). However, in each case, to assess the change in the amino acid profile, it is necessary to take into account the characteristics of the sample due to the high heterogeneity of breast cancer.

The construction of the ROC curve makes it possible to assess the classification accuracy for separating BC patients from healthy controls. According to Park et al. [53], for Lys, His, Thr, and Trp, the area under the curve varies in the range of 0.527–0.583; for Leu/Ile, the area is 0.728; for Phe, the area is 0.838; and for 5-hydroxyproline, the area is 0.968 on the training set. However, in the test set for 5-hydroxyproline, the area under the curve decreases to 0.744. With the simultaneous evaluation of many metabolites, which include amino acids, the accuracy of diagnosis increases. Thus, An et al. [56] showed that with the simultaneous evaluation of 47 metabolites using the random forest method, AUC values of 0.998 were obtained. Jasby et al. [6], using 30 metabolites, showed that AUC = 0.89 (95% CI: 0.85–0.93), sensitivity = 0.80, and specificity = 0.75. Baranovicova et al. [57] showed that AUC > 0.91 for 10 metabolites, while only for the BCAAs, AUC = 0.733. Only for the combination of 10 amino acids, Han et al. [58] showed that AUC = 0.91. Thus, individual amino acids have a low diagnostic value for the detection of breast cancer, while combinations of a number of amino acids with each other or with other metabolites provide a higher accuracy. However, in each specific case, it is necessary to check the data obtained during the construction of the model on a test sample in order to obtain an objective idea of the diagnostic capabilities of the algorithm.

### 2.2. Features of the Amino Acid Composition of Serum/Plasma in Different Molecular Biological Subtypes of Breast Cancer

In a number of studies, special attention was paid to the influence of the molecular biological characteristics of breast cancer on the amino acid profile of blood serum/plasma. Thus, Ala had a significant difference between ER-positive and ER-negative breast cancer [16,66]. Fan Y. identified a panel of eight potential low-molecular-weight biomarkers for diagnosing breast cancer subtypes, which included three amino acids: Pro, Ala, and Val [16]. Elevated Pro levels (FC = 1.217, *p* = 0.007) may indicate the suppression of proline oxidase in the human growth factor receptor 2 (HER2) positive group [67]. Ala was the most significantly reduced metabolite (FC = 0.544, *p* < 0.001) in the ER-positive participants compared to the ER-negative group [66]. Val was significantly increased in the HER2-positive groups compared to the HER2-negative groups, but was markedly decreased in the ER-positive groups compared to the ER-negative groups. Val anomalies indicated a violation of energy supply in the HER2-positive (fold change = 1.187, *p* = 0.002) and ER-positive (fold change = 0.682, *p* < 0.001) patients. The authors emphasized the clinical predictive potential of the identified eight biomarkers for breast cancer subtypes. An average prediction accuracy of 88.5% (95% CI 83.3–93.7%) was obtained for the training set and an average prediction accuracy of 85.6% (95% CI 80.9–90.1%) was obtained for the test set.

Han et al. [58] studied TNBC (tumor control) as well as the non-malignant pathologies of mammary glands. Four amino acids (Phe, Tyr, Ser, and Arg) were found to be more common in the triple-negative breast cancer (TNBC) group, while six amino acids (Pro, Trp, Val, Thr, Ala, and His) were more common in the benign group. The sensitivity, specificity, and accuracy of the classification model for identifying healthy controls and patients with TNBC were 91%, 100%, and 95%, respectively. Shen et al. observed eight amino acids whose levels were significantly lower in patients with triple-negative breast cancer than in healthy controls (Asp, Ala, His, Tyr, Trp, Met, Arg, and Pro), and two amino acids whose levels were significantly lower in patients with ER+/PR+ breast cancer than healthy individuals (Ala and His) [8].

Baranovicova et al. [57] showed that the levels of circulating metabolites are not associated with breast cancer molecular subtypes (luminal A/luminal B), histological findings, or stage. According to the authors, in the early stage of breast cancer, patients have common metabolic fingerprints in blood plasma, regardless of the degree, stage, or molecular subtype of breast cancer. However, statistically significant correlations were found between the level of tumor proliferation marker Ki-67 and circulating metabolites: Ala, Tyr, Gln, His, and Pro [57].

A significant decrease in the Trp concentration was observed in the plasma of luminal A, triple-negative, and HER2-positive breast cancer compared with healthy controls [68].

### 2.3. Racial Characteristics of the Serum/Plasma Amino Acid Profile in Breast Cancer

Breast cancer is associated with marked metabolic changes. However, these metabolic shifts in tumors may differ between stages, subtypes, and race [8,69]. Racial differences may not only influence metabolite levels, but also modify associations between metabolites and breast cancer. Racial differences in the incidence of breast cancer are well documented [70,71,72]. Compared to Caucasian American (CA) women, African American (AA) women tend to develop more aggressive tumors that are characterized by an earlier age at diagnosis, a higher mitotic index, and a lower prevalence of ER/PR expression, and subsequently have a lower survival rate. It has been suggested that genetic predisposition plays a role in racial/ethnic disparity in breast cancer [73,74]. For example, Gieger et al. found that up to 12% of the observed dispersion of the metabolic homeostasis of the human body could be explained by genetic variants [75].

In a study by Shen et al., when stratified by race, the difference in the plasma amino acid composition between healthy and sick patients was more evident in the AA participants than in the CA participants [8]. However, due to the small sample size, no significant difference remained in either the AA or CA participants after adjusting for multiple comparisons [8]. A study by Cala et al. described the metabolic features in breast cancer in Latin woman of Hispanic origin [33].

Santaliz Casiano et al. found that amino acid levels are significantly lower in AA breast cancer patients than in healthy individuals [59]. The pathways associated with energy metabolism are glycolysis, amino acid metabolism, and the TCA cycle, which dominate in AA women with ER+ tumors, potentially indicating the aggressiveness of their tumors [76]. In AA individuals, metabolites associated with aminoacyl-tRNA biosynthesis, Arg metabolism, branched amino acid metabolism, and His metabolism were differently distributed in the plasma in individuals with breast cancer. It is known that tumor cells need amino acids both as alternative fuel and for DNA synthesis, building new blood vessels and supporting their rapid growth and proliferation. Amino acids provide metabolic intermediates for epigenetic regulation [77]. In particular, Met levels were found to be lower in plasma samples from AA women who had breast cancer. Met is a methyl group donor for methylation and is a major contributor to epigenetic regulation [78,79]. Systemic or cellular metabolic changes affect the epigenetic landscape, which is important for ERα activity and the response to clinical drugs [80,81]. In AA women, poverty rates are correlated with the hypermethylation of cancer-associated pathways, including the glucocorticoid receptor, p53, estrogen-dependent breast cancer signaling, and cell proliferation [82]. Therefore, hypermethylation is a possible biological mechanism that may explain the poorer outcomes in AA women with live-caused breast cancer in areas of low socioeconomic status.2.4. Serum/Plasma Amino Acids in Breast Cancer Risk Assessment.

Amino acids are most often positively associated with breast cancer risk, and among them are the branched-chain amino acids Val and Leu, as well as Lys, Arg, Phe, and Gln [83]. They are also positively associated with the risk of His as a necessary precursor of histamine, the release of which is an early event in inflammatory responses and is a regulator of cell proliferation [84].

The branched-chain amino acids (BCAAs) Leu, Val, and Ile are dietary essential amino acids and are important metabolites that are involved in cell signaling pathways and muscle protein synthesis [85]. Elevated plasma BCAA concentrations are strongly positively correlated with body mass index and insulin resistance and are markers of a dysfunctional metabolism [86]. In premenopause, elevated levels of circulating BCAAs have been associated with a lower risk of breast cancer (Table 3) [86]. In contrast, among postmenopausal women, elevated levels of circulating BCAAs have been associated with an increased risk of breast cancer [87].

Several studies have evaluated the effects of BCAAs on breast cancer risk with conflicting results, and only one study has assessed menopausal status [90,94,95]. A survival analysis showed that the expression of the catabolic BCAA gene is closely associated with long-term oncological outcomes [96]. High BCAA levels suppressed both tumor growth and breast cancer metastasis, demonstrating the potential benefits of increasing dietary BCAA intake during breast cancer therapy [97,98]. After stratification based on menopausal status, there was a significant inverse relationship between BCAA intake and the likelihood of postmenopausal breast cancer (RR = 0.22; 95% CI 0.13–0.39), although this significant association was not found in premenopausal breast cancer (RR = 2.57, 95% CI 0.51–12.73) [99].

Jobard et al. showed that in the premenopausal subgroup, breast cancer can be predicted by several risk-related metabolites, including His with moderate accuracy (AUC = 0.61, 95% CI: 0.49–0.73) (Table 3) [92]. Predictive power or significant metabolites have not been found in general or in postmenopausal women. Lecuyer et al. found high levels of Gln, Arg, Lys, and Val to be closely associated with a higher risk of BC [89]. Another study reported that higher levels of Gln/isoglutamine, Val/norvaline, Trp, and Phe were related to an increased risk of BC [95]. A study by Nagata et al. showed that the plasma levels of certain specific amino acids, such as Arg, Leu, Tyr, and Asp, were associated with endogenous sex hormone levels, sex-hormone-binding globulin (SHBG), or insulin-like growth factor (IGF-1), as determined by biomarkers of breast cancer risk [88].

Stevens et al. [93] found that Gln was associated with a reduced risk of breast cancer, as in studies involving pre- and postmenopausal women in EPIC [93], but other studies produced conflicting results. Gln was associated with an increased risk in the SU.VI.MAX [95] and E3N [92] cohorts, where the association was limited to premenopausal women. Mrowiec et al. [100] showed that the serum Asn concentration was significantly higher in late-detected breast cancer compared with early-detected breast cancer only in a subgroup of older women. The authors found that several metabolic pathways, including BCAA degradation and glutathione metabolism, differed between younger and older women with a cut-off point of 45 years. The overrepresentation of pathways associated with metabolites that differentiate early- and late-diagnosed cancer (His and Ala metabolism) was observed only in a subgroup of older women, which, once again, confirmed the age-related nature of the metabolic features associated with the risk of developing breast cancer. The results of His et al. [90] show that the concentration of amino acids in plasma is inversely related to the risk of breast cancer; however, these results contradict those of other studies, where, on the contrary, an increase in the concentration of amino acids contributed to an increase in the risk of breast cancer (Table 3).

Risi et al. conducted a study on the risk of breast cancer recurrence [101]. Higher levels of Phe and lower serum levels of Leu, Ile, Val, and His were shown to be associated with the presence of advanced breast cancer. Despite the fact that no statistically significant correlation was found between the levels of individual metabolites and the risk of recurrence in the entire cohort of breast cancer patients, Phe was significantly associated with advanced breast cancer that increased with breast cancer recurrence. However, the authors proposed a metabolomics model that demonstrated strong predictive power. Patients with a “high risk” had a significantly increased likelihood of disease recurrence compared to patients with a low-risk metabolomics fingerprint (HR = 3.42, 95% CI 1.58–7.37, *p* < 0.001). These results are consistent with those of other studies [102].

The AminoIndex Cancer Screening (AICS) technology has been described and used as a new cancer risk calculation method for early cancer diagnosis [34,49,103,104,105]. AICS (breast) detects breast cancer by detecting abnormal plasma concentrations of Thr, Ala, Orn, His, and Trp [106]. In breast cancer, the Thr, Ala, and Orn concentrations are elevated, while the His and Trp concentrations are reduced, and a multivariate analysis shows an overall accuracy of 88.2% [107].

Thus, it was shown that the risk of breast cancer is associated with the amino acid composition of blood plasma/serum; however, the patterns identified by the authors are closely related to the characteristics of the sample, menopausal status, and age. Nevertheless, according to most authors, Gln/Glu, Asp, Arg, Leu/Ile, Lys, and Orn have the greatest significance in assessing the risk of breast cancer.

## 3. Amino Acid Metabolism in Breast Cancer

### 3.1. Metabolic Features of Breast Cancer

The amino acid metabolism plays a critical role in the proliferation of breast cancer cells [108,109]. The intake and use of amino acids helps to support the growth of cancer cells [110,111,112]. A number of studies have shown that a decrease in the content of a number of amino acids may be the result of their excessive consumption or preferential use to support the uncontrolled growth of breast cancer cells [5,26,37,111,112,113,114].

The Glu/Gln, Ala, Asp, and Arg metabolisms were the most important biosynthetic pathways in breast cancer, suggesting extensive metabolic disturbances during breast cancer progression [115,116]. Huang S. et al. showed that the alanine, aspartate, and glutamate pathways are critical biological pathways for the early diagnosis of breast cancer [30]. Most of the metabolites in these three metabolic pathways were reduced in breast cancer patients compared to healthy controls. Thus, Ala promotes the proliferation of breast cancer cells, which indicates the potential role of Ala as a marker for cancer diagnosis [52]. The down-regulation of Gln indicated that Glu can accumulate in the body, which contributes to the development of breast cancer due to the increased proliferation of mammary epithelial cells [117] due to ATP production and nucleotide biosynthesis [118]. Moreover, Glu activation via glutaminolysis can maintain the citric acid cycle [119]. The metabolism of Gln is closely related to the process of providing energy to the cancer cell. Gln is transported into cancer cells by means of multiple transporters [116,120]. It was previously shown that the expressions of the Gln transporters ASCT2, SNAT1, SNAT2, and SNAT5 are increased in tumor tissue [121]. On the other hand, the inhibition of ASCT2 reduces the growth of TNBC [122]. It is known that the reductive metabolism of Gln supports tumor growth under conditions of hypoxia, mitochondrial dysfunction [123], and in an environment with a low nutrient content [124]. It was noted that Gln/Glu reversibility decreased in MCF-7 cells, indicating that breast cancer cells may be partially associated with irreversible glutaminase [125]. The Gln/Glu ratio can be used as a biomarker for the diagnosis of breast cancer [126]. The enzyme glutaminase I (GLS-I), which converts Gln to Glu via glutaminolysis, can be considered as a target for breast cancer therapy [127].

The change in the Gln level can be reflected in fluctuating levels of Ala and Asp due to the abnormal transport of ammonia. Higher histidine decarboxylase activity may lead to a decrease in the His levels [128], and low concentrations of His may be associated with increases in Asp and Glu, which can be converted to oxaloacetic and α-ketoglutaric acids, which are intermediates of the tricarboxylic acid cycle. Asp has been shown to be more sensitive to breast cancer [129]. Therefore, an increase in the use of Asp by BC cells can lead to a decrease in the levels of Asp and oxaloacetate in the blood. It is noteworthy that, as a transamination product of aspartic acid, Asp has an important effect on breast cancer metastasis [130].

The dysregulation of branched-chain amino acid (BCAA) metabolism, including Leu, Ile, and Val, has been reported to be associated with specific cancer phenotypes. BCAAs can inhibit tumor growth and metastasis [97], so changes in the BCAA levels can often reflect systemic changes in cancer patients compared to healthy controls [131]. Plasma Arg, Pro, and Trp metabolites decreased in BC patients [6]. Huang et al. [30] revealed a decrease in the Ser and Thr levels in the serum of patients with breast cancer. Harvie et al. showed that a Tyr deficiency can lead to the stunting of breast cancer cells [132], and the inhibition of tumor growth was confirmed with diets that were low in Phe and Tyr in an animal study [133]. It is known that sharp metabolic shifts in the levels of choline and Pro are characteristic of metastatic breast cancer [134]. Breast cancer has been shown to be highly dependent on Arg [6]. It has been shown to enhance the immune response, both innate and adaptive, with the administration of Arg supplements [135]. Conversely, a decrease in the dietary Asn intake or the suppression of asparagine synthetase reduced breast cancer metastasis [130].

Many authors agree that the concentration of Trp in the plasma and serum of breast cancer patients is reduced [26,28,113]. Previously, it was shown that Trp indirectly promotes the degradation of the extracellular matrix and the invasion of cancer cells [136]. Two major enzymes catalyze Trp into metabolites of the kynurenine (Kyn) pathway: indolamine 2,3 dioxygenase (IDO1) and tryptophan 2,3 dioxygenase (TDO2) [137]. Kyn activates the aryl hydrocarbon receptor, which promotes the evasion of the cancer immune response by increasing IL-10 and suppressing immune activation cells [138]. Thus, with IDO1/TDO2 overexpression, increased Trp catabolism can lead to a decrease in its serum concentration and an accumulation of Kyn metabolites [139].

Ser is transported into cells by means of ASCT1, which is highly expressed in breast cancer [140]. An increase in the rate of tumor cell proliferation depends on the presence of extracellular Ser. In an experiment with mice, it was shown that decreases in the Ser and Gly levels suppresses tumor growth and increases the lifespan [141]. It is interesting to note that, depending on the type of cancer, either Ser or Gly can contribute to the rapid proliferation of cancer cells [142].

Budhu A. et al. showed that a decrease in plasma Cys was inversely associated with an increase in Cys in breast cancer tissues [13], suggesting that breast cancer cells use more Cys. Cys is involved in the redox reaction of glutathione. With an increase in the concentration of Cys, oxidative damage and the production of free radicals also increase, which leads to gene mutation [143]. On the other hand, Cys can be considered as a substrate for the production of hydrogen sulfide, which stimulates cellular bioenergetics [144]. It has been established that the Cys-associated metabolic pathway of cysteinyl leukotrienes (CysLT) is also closely associated with cancer in enhancing the ability to survive and proliferate cancer cells [145].

### 3.2. Amino Acid Metabolism as a Target for Breast Cancer Imaging

Elevated levels of Met, Gln, Cys, Trp, Tyr, and other amino acids in tissues have been noted in many malignancies, including breast cancer [146]. Cancer cells with the up-regulation of amino acid metabolism stimulate the increased transport of amino acids into the cell [147]. The increased consumption of amino acids and the overexpression of amino acid transporters in malignant tumors make radiolabeled amino acids attractive imaging agents [114,148].

Multiple amino acid transporters have been demonstrated to be up-regulated in breast cancer families, including L-type amino acid transporter (LAT1), ASC transporter 2 (ASCT2), ATB^0,+^, SNAT1, and xc- [149,150]. LAT1 is required for the transport of large neutral amino acids and is overexpressed in many types of malignancies, including breast cancer [151]. The expression of ASCT2 also has prognostic associations in breast cancer [120]. The xc-system transporter, which mediates cysteine uptake, is up-regulated in some breast cancer tumors [152]. The two amino acid transporters SLC7A5 and SLC7A11 are considered essential for the growth of breast cancer cells in a cell-dependent manner [153].

Met is a naturally occurring large neutral amino acid that is readily labeled with the 11C radioactive isotope. ^11^C-methionine serves as a metabolic marker for Met uptake via L-type amino acid transporters. PET with ^11^C-methionine makes it possible to visualize primary and metastatic lesions, as well as to predict the response to breast cancer treatment [154]. Limitations of ^11^C-methionine include the ability to detect metastases only in the liver and bone marrow and its relatively short half-life (20 min). On the basis of Met, an MR contrast agent based on Met-MSN-Gd^3+^ was developed that targets methionine receptors, which are overexpressed in tumor cells (MSN—Mesoporous Silica Nanoparticles) [155,156].

*Trans*-1-amino-3-^18^F-fluorocyclobutanecarboxylic acid (anti-^18^F-FACBC, also known as ^18^F-fluciclovine) is a synthetic analogue of Leu, which is transported into the cell by the ASCT2 transporter with the additional involvement of LAT1 [157]. Uptake by cells is most similar to uptake of the natural amino acid Gln [158]. ^18^F-fluciclovine can visualize breast lesions [159,160], axillary lymph node metastases [161], and previously undetected extra-axillary nodular metastases [161,162]. Preclinical studies have demonstrated significant success with 18F-fluciclovine in detecting bone metastases.

(4S)-4-(3-[^18^F]fluoropropyl)-l-glutamate (BAY 94-9392, also known as [^18^F]FSPG), is a synthetic amino acid analogue of SLC7A11 [152,163]. Histological or molecular subtypes of breast cancer may affect ^18^F-FSPG uptake. ^18^F-5-fluoroaminosuberic acid, a synthetic amino acid substrate of SLC7A11, exhibited tumor uptake in three breast cancer cell lines (MDA-MB-231, MCF-7, and ZR-75-1), with the highest uptake observed in MDA-MB- 231, the TNBC cell line [164].

Several radioactively labeled Tyr analogues have been developed for tumor imaging, including L-[1–^11^C]tyrosine [165]. Technetium-labeled tyrosine analogs have also been synthesized in high yield and can distinguish malignant breast neoplasms from benign breast tissues [166]. O-(2-^18^F-fluoroethyl)-l-tyrosine (^18^F-FET) is a synthetic amino acid transported by SLC7A5 [167]. Animal experiments using rats and mice have shown that ^18^F-FET can distinguish between inflammation and malignancy [168,169] as well as in 75% of breast cancer patients.

In preclinical studies, several propanoic acid derivatives have demonstrated good tumor uptake in human breast cancer cells as well as mouse tumor xenografts [170]. 2-Amino-5-(4-[^18^F]fluorophenyl)pent-4-ynoic acid ([^18^F]FPhPA) is a synthetic amino acid that targets SLC1A5 and SLC7A5. A high uptake of the radiopharmaceutical [^18^F]FPhPA was detected in the mouse breast cancer cell line EMT6 by PET [171]. Trp analogs, primarily using L-type amino acid transport, have been developed by several research groups and have also been shown to be taken up by breast cancer cells in small animal studies [172]. An analogue of Leu, 5-[^18^F]fluoroleucine, was synthesized with primary transport via LAT1 [173]. Unlike ^18^F-fluciclovine, the absorption of 5-[^18^F]fluoroleucine gradually increases over time. The glutaminolysis pathway is very active in many malignancies, including triple-negative breast cancer. Preclinical work with the Gln analog [^18^F](2S,4R)4-fluoroglutamine demonstrated the ability of this indicator to track changes in the size of the cellular Gln pool and the glutaminolysis pathway after glutaminase inhibition [174].

Amino acid transporters can also be used to image tumors using single-photon emission computed tomography (SPECT). 3-[^123^I]-α-methyl-l-tyrosine (IMT) is an artificial amino acid that is transported through SLC7A5 [175] and is also a suitable metabolic indicator for SPECT in extracranial tumors, including breast cancer [176]. Using IMT SPECT, primary and metastatic breast cancer, as well as tumor regression after radiotherapy, were detected and were consistent with the results of the clinical assessment [175]. [99m]Tc-labeled diethylenetriaminepentaaceticacid (DTPA-bis)-methionine scintymammography has shown 96% sensitivity and a 96% positive predictive value for the detection of breast cancer [177], and therefore may be an alternative to conventional SPECT using non-specific mitochondrial uptake.

## 4. Amino Acids in Potential Strategies for the Treatment of Breast Cancer

One of the therapeutic approaches to cancer treatment is aimed at changing the metabolism of the tumor [178,179].

Some essential amino acids (EAAs)—Trp, His, Met, and branched-chain amino acids—are directly associated with tumor growth and treatment resistance [180,181,182,183]. EAAs provide important raw materials for protein synthesis, influence the biological behavior of cells, and induce therapeutic resistance in breast cancer cells. Strekalova et al. [184] found that Met plays a critical role in maintaining the self-renewal and survival of cancer stem cells. Met restriction reduced the population of cancer stem cells in TNBC. Saito et al. [185] showed that Leu not only promotes cell proliferation in ER-positive breast cancer, but also participates in the mechanism of resistance to tamoxifen. In addition, essential amino acids, as important nutrients, mediate the development of an immunosuppressive tumor microenvironment in breast cancer. For example, kynurenine, a product of Trp metabolism, can inhibit T cell proliferation and differentiation, leading to immune evasion and tumor progression in breast cancer [186]. Interestingly, both Trp and kynurenine were lower in plasma in breast cancer patients compared to controls, especially in women with estrogen-receptor-negative and advanced breast cancers [187]. These results show an intrinsic relationship between EAA metabolism and the immune microenvironment in breast cancer. Zhao et al. [188] found that the ratio of SLC7A5 to SLC7A8 (SSR) is significantly correlated with the level of EAA and the metabolic activity of EAA in breast cancer, and therefore, the SSR index can be used as a biomarker to assess the degree of metabolism of EAA in breast cancer. In addition, breast cancer patients with a high EAA metabolism had a shorter overall survival time, a higher PD-L1 expression, and higher T-regulatory cell infiltration, indicating that a high EAA metabolism was associated with a poor prognosis and immunosuppression in breast cancer.

Zhang, L. showed that the level of BCAA in the plasma and cancer tissues of BC patients was increased, which was accompanied by an increase in the expression of BCAA catabolism enzymes [189]. The stimulation of BCAA catabolism by modulating BCAT1 enhanced the growth and formation of colonies of breast cancer cells. BCAT1 promoted mitochondrial biogenesis and enhanced mitochondrial function by promoting ATP production and protection against oxidative stress by activating mTOR signaling, but not AMPK or SIRT1. The inhibition of mTOR by rapamycin neutralizes the role of BCAT1 in mitochondrial function and cancer cell growth.

For some cancer subtypes, such as TNBC, there is no specific therapy, resulting in a poor prognosis that is associated with invasion and metastasis.

Under physiological conditions, Gln is transported into cells by many transporters such as Ala, Ser, Cys-preferential transporter 2 (ASCT2, also known as SLC1A5), and L-type amino acid transporter 1 (LAT1, also known as SLC7A5). In TNBC, both ASCT2 and LAT1 are overexpressed [122,190]. Compared to other subtypes of breast cancer, TNBC is more Gln dependent and sensitive to glutaminolysis-targeted therapy due to glutaminase overexpression (GLS) [191,192], which is associated with high-grade metastatic breast cancer. Several small-molecule GLS inhibitors, such as CB-839, have been developed to combat the dysregulation of glutaminolysis [193]. Other combination therapies, such as the combination of GLS inhibition and bevacizumab (an anti-angiogenesis monoclonal antibody targeting VEGF), also show antitumor effects on TNBC [194]. Ginsenoside was described to effectively inhibit TNBC by suppressing Gln uptake and Glu production by down-regulating glutaminase 1 (GLS1) expression [195]. Ginsenoside treatment further reduced cellular ATP production, decreased amino acid utilization associated with Gln metabolism, and induced glutathione depletion and reactive oxygen species accumulation, which consequently triggered apoptosis in TNBC. Morotti et al. showed that the knockdown of the Gln transporter SLC38A2, which was identified as a highly expressed amino acid transporter in six breast cancer cell lines [196], reduced Gln uptake, inhibited cell growth, induced autophagy, and resulted in the production of reactive oxygen species in a subgroup of Gln-sensitive cell lines. A high expression of the SLC38A2 protein was associated with poor breast cancer survival in a large group of patients (*p* = 0.004), especially in TNBC (*p* = 0.02).

Increased Glu production by GLS may support the uptake of exogenous cystine via the cystine/glutamate antiporter xCT to maintain redox balance. As a clinically approved anti-inflammatory drug, sulfasalazine (SASP) was found to inhibit xCT activity and retard the growth of TNBC [197]. By immunizing mice with a DNA-based vaccine expressing the xCT protein, the cell surface immunotargeting of the xCT antigen effectively attenuated tumor growth and lung metastasis, and increased chemosensitivity to doxorubicin [198]. Additionally, virus-like particle immunotherapy was developed, which elicits a stronger humoral response against xCT [199].

In addition to Gln and Cys, TNBC cells are also somewhat dependent on the availability of several other amino acids such as Met, Asp, and Arg, suggesting that restricting these amino acids may have a therapeutic effect [200]. The depletion of either Met or Gln can increase the cell surface expression of the pro-apoptotic TNF-related apoptosis-inducing ligand receptor-2 (TRAIL-R2) and increase the sensitivity of TNBC cells to TRAIL-induced apoptosis [130,201]. Met deprivation in the diet increases cellular susceptibility to lexatumumab, an agonistic monoclonal antibody targeting TRAIL-R2, and reduces the rate of lung metastasis [202]. In addition, many tumor and stem cells depend on the biosynthesis of the universal methyl donor S-adenosylmethionine from the exogenous Met via methionine adenosyltransferase 2α (MAT2A) to maintain their epigenome [203,204]. A restriction on Met is enough to undermine the ability of TNBC to initiate a tumor, which is in part due to the impaired formation of S-adenosylmethionine. The combination of methionine restriction and the MAT2A inhibitor cycloleucine has a synergistic antitumor effect [184]. Under normal physiological conditions, the serum levels of Asp are lower than in the mammary gland, making Asp bioavailability a key regulator of circulating tumor cells and the metastatic potential of breast cancer. The restriction of Asp intake by the suppression of asparagine synthetase, treatment with L-asparaginase, or the restriction of Asp intake in the diet inhibits breast cancer metastasis [201]. The depletion of Arg by recombinant human arginase (rhArg) leads to the apoptosis of TNBC cells via reactive oxygen species and induces adaptive autophagy, while blocking the flow of autophagy via autophagy-targeting drugs enhances rhArg cytotoxicity [205]. The deprivation of Arg by L-arginase impairs tumor growth, leading to cell death [206]. Much attention has been paid to epigenetic modifications caused by enzymes of protein arginine methyltransferases, in which methylate Arg make a great contribution to the process of breast carcinogenesis and tumor suppression [207] and are targets for many types of cancer [208].

Leu uptake is predominantly mediated by the L-type amino acid transporter (LAT) family, a group of four Na+-independent transporters (LAT1, SLC7A5; LAT2, SLC7A8; LAT3, SLC43A1; and LAT4, SLC43A2) with an affinity for branched and neutral amino acid transporters [130]. Glutamine transport is largely mediated by Ala, Ser, and Cys-preferential transporter 2 (ASCT2; SLC1A5). The authors proposed using the ASCT2 inhibitor, benzylserine (BenSer), to doubly inhibit Gln [209] and Leu [210] uptake. It has been shown that a double inhibition with the pharmacological inhibitor BenSer can reduce the growth of breast cancer cells and limit the progression of the cell cycle.

Amino acid deprivation (AADT) is becoming a promising strategy for the development of new therapeutic agents against cancer [211]. The rapid growth of tumors leads to a decrease in the expression of certain enzymes, which leads to the auxotrophy of some specific amino acids. Amino acid depletion selectively inhibits tumor growth because normal cells can synthesize amino acids through their normal machinery. The enzymes used in AADT are primarily obtained from microbes due to their easy availability. Thus, the deprivation of Gln leads to a decrease in cell proliferation and cell death in breast cancer cell lines [212].

Endocrine therapy is the standard treatment for estrogen-receptor-positive (ER+) breast cancer, but 40% of women experience a recurrence of the disease during therapy. A general analysis of transcription in cells revealed a suppression of the neutral and basic amino acid transporter SLC6A14, which is regulated by the increased expression of miR-23b-3p, which leads to impaired amino acid metabolism [213]. This altered cellular amino acid metabolism is supported by autophagy activation and the increased import of acidic amino acids (Asp and Glu) mediated by the SLC1A2 transporter. Targeting these amino acid metabolic dependencies increases the sensitivity of cells to endocrine therapy.

Anticancer agents delivered to cancer cells often exhibit multidrug resistance due to a displacement of the agents. One way to solve this problem is to increase the accumulation of anticancer agents in cells with the help of amino acid transporters. Val-lapatinib and Tyr-lapatinib were newly synthesized by adding Val and Tyr fragments, respectively, to the parent anticancer agent lapatinib. Val-lapatinib and Tyr-lapatinib demonstrated enhanced anticancer activity compared to parental lapatinib in various cancer cell lines (MDA-MB-231 and MCF7) [214]. Both Val-lapatinib and Tyr-lapatinib, but not the parent lapatinib, inhibit glutamine transport in MDA-MB-231 and MCF7 cells, suggesting the involvement of amino acid transporters. Thus, amino acid transporters can be effective drug delivery targets to increase the uptake of anticancer agents, leading to one method of overcoming multidrug resistance in cancer cells.

Mello-Andrade et al. studied the effect of ruthenium(II) complexes associated with the amino acids methionine (RuMet) and tryptophan (RuTrp) on the induction of cell death, clonogenic survival, the inhibition of angiogenesis, and the migration of MDA-MB-231 cells [215]. The study also showed that RuMet and RuTrp complexes induce cell cycle arrest and the apoptosis of MDA-MB-231 cells, as evidenced by an increase in the number of annexin V-positive cells, p53 phosphorylation, caspase 3 activation, and poly(ADP-ribose) polymerase cleavage. RuMet and RuTrp complexes act directly on breast tumor cells, leading to cell death and suppressing their metastatic potential; this reveals the potential therapeutic effect of these drugs.

The use of various drugs based on platinum nanoparticles leads to a disruption of the amino acid metabolism, a disruption of tRNA aminoacylation, and protein synthesis [216]. Mitrevska et al. [217] analyzed the effect on amino acid metabolism in MDA-MB-231 cells upon treatment with cisplatin, PtNP-10, and PtNP-40, and revealed a marked contrast between the effects of cisplatin and PtNP. The results indicate a higher sensitivity of MDA-MB-231 cells to PtNP compared to cisplatin, since the increase in the number of amino acids was associated with the degree of insensitivity to various chemotherapeutic agents [217].

In general, combinations of anticancer drugs and amino acids can improve the intratumoral distribution of the active substance and increase its bioavailability. In particular, amino acid-based poly(ether urea ester) (AA-PEUU), as a nanocarrier for the systemic delivery of gamboginic acid, demonstrates the effectiveness of engineered AA-PEUU nanocarriers with custom structures and universal customization for the systemic delivery of therapeutics in the treatment of TNBC [218].

Plasma amino acid analysis can be used to monitor treatment progress. So, Minet-Quinard et al. [47] showed that the plasma levels of Ser and Glu returned to normal six months after the surgical removal of the tumor. Dunstan et al. [219] considered the changes in the amino acid homeostasis during radiation therapy. The urinary histidine and alanine levels were shown to be elevated prior to radiotherapy, while the Thr, Met, Ala, Ser, Asp, and Gln levels were higher after 5 weeks of radiotherapy. Many complications such as cachexia, anorexia, and fatigue occur in the treatment of problems associated with breast cancer, and many studies have considered the addition of amino acids with BCAAs [220] or a single amino acid or its derivative [221] to reduce the effects of treatment. Li et al. provided preliminary data to support the correction of the Trp metabolism for the treatment of neuropsychiatric symptoms [222].

Changes in the concentration of metabolites may also be useful in predicting the overall survival of patients with breast cancer. Thus, two metabolites differ significantly depending on the previous therapy: Met and Ser [223]. The blood Met levels were higher in the patients treated with anti-Her2 therapy, while the Ser levels were lower in the patients treated with endocrine therapy alone. Patients with TNBC were previously shown to have higher Ser levels, while patients with luminal cancer A, on the contrary, had low blood Ser concentrations [224], which is consistent with the therapy and Ser accumulation with anti-Her2 therapy [225]. In addition, Possemato et al. found an increased flux of Ser synthesis in patients with estrogen-negative breast cancer, which is also associated with a poor 5-year survival [226].

## 5. Conclusions

A fairly large number of studies have been devoted to the study of amino acid metabolism in breast cancer. At the same time, both the contents of individual amino acids and the combinations of amino acids with each other or with other metabolites determined in the course of obtaining the metabolomic profiles of biological fluids were determined. We have shown that for some amino acids (Thr, Arg, Met, and Ser) an increase in concentration is more often observed in breast cancer compared with a healthy control, and for other amino acids, there is a decrease (Asp, Pro, Trp, and His). However, the amino acid profile must be analyzed while taking into account the high heterogeneity of breast cancer, as well as age and race. The accuracy of the diagnosis using amino acids depends on the number of metabolites in the algorithm and varies from 52 to 98%. The contents of amino acids in biological fluids are used to assess the risk of breast cancer; however, the identified patterns are closely related to the characteristics of the sample, menopausal status, and age. According to most authors, Gln/Glu, Asp, Arg, Leu/Ile, Lys, and Orn have the greatest significance in assessing the risk of breast cancer. An analysis of the changes in the amino acid metabolism in breast cancer is a promising strategy for developing new therapeutic agents, monitoring the treatment process, correcting complications after treatment, and evaluating the survival rates. This, once again, emphasizes the high importance of research in this area.

## Figures and Tables

**Table 1:** Basic studies of the amino acid composition of plasma/serum in breast cancer.

No.	Author, Year	BC/Control	BC Stages	Method	Up-Regulated AAs	Down-Regulated AAs
1	Kubota A., 1992 [44]	22/11	St I + II—22	AA analyzer	Ala, Arg, Thr	Cys, Gln
2	Cascino A., 1995 [45]	33/28	-	AA analyzer	Glu, Orn, Trp	-
3	Proenza A.M.A., 2003 [46]	16/18	St I—2, St II—5, St III—3, St IV—5, Unknown—1	HPLC	Asn, Gln, Pro (Hydroxyproline)	Asp
4	Minet-Quinard R., 2004 [47]	19/18	T0—16%, T1—42%, T2—42%	AA analyzer	Ser, Glu, Orn	-
5	Vissers Y.L.J., 2005 [48]	22/22	St I—6, St II—7, St III—8	HPLC	-	Arg
6	Okamoto N., 2009 [49]	61/51	St 0—8, St I—30, St II—18, St III–5	HPLC–ESI–MS	Thr, Ser, Glu, Orn	Met, Ile, Phe, Arg
7	Miyagi Y., 2011 [34]	196/976	St II—95, St III—19, St IV—15, Unknown—5	HPLC–ESI–MS	Thr, Pro, Ser, Gly, Ala, Orn	Gln, Trp, His, Phe, Tyr
8	Shen J., 2013 [8]	60/60	ER+/PR+—30, triple negative—30 African Americans and Caucasian Americans data sets	UPLC-MS/MS or GC-MS	-	Ala, His, Asp, Lys, Tyr, Trp, Met, Arg, Pro
9	Poschke I., 2013 [50]	41/9	-	HPLC	Glu, Ser, Gln, Ala, Val, Phe, Ile, Leu	-
10	Barnes T., 2014 [51]	8/8	St I + II—8	HPLC	Ala, Asp, Gln, Lys, Met, Tyr	Arg, Gly, Pro, Ser, Ile, Val, Orn
11	Gu Y., 2015 [52]	28/137	St I—7, St II—18, St III—3	AA analyzer	Thr, Arg	Asp, Gln, Gly, His
12	Jové M., 2017 [5]	91/20	St I—2, St IIA—40, St IIB—30, St IIIA—13, St IIIB—7	ESI-Q-TOF MS/MS	-	Gln, Arg, Lys, Val
13	Wang X., 2018 [7]	44/34	-	UPLC-MS	Ala, Asp, Cys, Gly, Glu, Gln, His, Ile, Met, Pro, Phe, Ser, Tyr, Val	Arg
14	Jasbi P., 2018 [6]	102/99	St I—24, St II—42, St III—36	UPLC-MS	Asp	Pro
15	Eniu D.T., 2018 [28]	30/26	St I—3, St II—17, St III—10	HPLC-MS	-	Tyr, Arg, Ala, Ile, Trp, Leu
16	Cala M.P., 2018 [33]	29/29	St I—3, St II—15, St III—11 Colombian Hispanic data set	GC-MS	Val, Ala, Ile, Ser, Glu, 4-Hydroxyproline	-
17	Park J., 2019 [53]	40/30	St I—15, St II—15, St III—10	HPLC-MS	5-oxoproline, Phe, Ile + Leu	-
18	Li L., 2020 [54]	105/35	St I—65, St II—40	NMR spectroscopy	Leu, Phe	Arg, Glu, Lys, Tyr, His
19	Politi C., 2021 [55]	38/10	-	GC-MS	Glu, Ile, Leu, Phe, Pro, Ser	Cys
20	An R., 2022 [56]	75/20	St I—31, St II—33, St III—11	UPLC-MS	Gln, Arg	Glu, Asp, Cys
21	Baranovicova E., 2022 [57]	50/46	St I + II—50	NMR spectroscopy	-	Leu, Ile, Val, Ala, His
22	Han X., 2022 [58]	30/30	ТНРМЖ	MALDI-TOF-MS	-	Ala, Ser, Pro, Val, Thr, His, Phe, Arg, Tyr, Trp
23	Santaliz-Casiano A., 2023 [59]	103/150	ER + breast cancer African American (AA) and non-Hispanic White (NHW) data sets	GC-MS	-	Arg (AA), His (AA), Met (AA)
24	Panigoro S.S., 2023 [60]	29/28	St I—13, St II—13, St III—3	HPLC	Cys	Glu, His, Orn, Thr, Tyr, Val

Note: HPLC—high performance liquid chromatography; GC-MS—gas chromatography coupled to a mass spectrometer; MALDI-TOF-MS—matrix-assisted laser desorption/ionization time-of-flight mass spectrometry; HPLC-ESI-MS—high performance liquid chromatography–electrospray ionization-mass spectrometry; UPLC-MS—ultra-performance liquid chromatography coupled to a mass spectrometer; UPLC-QTOF-MS—ultra-performance liquid chromatography coupled with quadrupole/time-of-flight mass spectrometry; NMR—nuclear magnetic resonance spectroscopy.

**Table 2:** Fold change BC/control according to different authors.

Amino acid	Kubota, 1992 [44]	Proenza, 2003 [46]	Minet-Quinard, 2004 [47]	Vissers, 2005 [48]	Okamoto, 2009 [49]	Miyagi, 2011 [34]	Shen, 2013 [8]	Barnes, 2014 [51]	Gu, 2015 [52]	Wang, 2018 [7]	Eniu, 2018 [28]	Cala, 2018 [33]	Park, 2019 [53]	Baranovicova, 2022 [57]	An, 2022 [56]	Panigoro, 2023 [60]
Ala	1.96	0.93	1.11	0.94	1.06	1.08	0.82/0.80 *	1.16	0.93	1.11	0.59	1.25		0.80	0.92	0.88
Arg	1.33		0.86		0.84	0.98	0.90/0.86	0.91	1.78	0.81	0.32				2.35	0.99
Asn		1.54	0.95	0.91	0.97	0.99	0.84/0.82	1.16		1.23	0.65				0.91	
Asp		0.45	0.80					1.33	0.67		0.71				0.43	
Cys	0.58						0.93/1.01		1.04	1.31	0.97				0.66	1.35
Gln	0.73	1.16	1.05	0.92	1.00	0.97	0.94/1.00	1.08		1.53	0.77				1.21	
Glu		1.02	1.42	1.24	1.40		1.05/1.01		0.46	1.81	0.42	1.27			0.58	0.86
Gly		0.94		1.01	1.08	1.12	0.90/0.91	0.91	0.90	1.16	0.68				0.94	
His	1.08	1.04	1.17	1.04	0.95	0.97	0.88/0.91		0.87	1.20	0.61		0.99	0.63	0.93	0.75
Ile		0.94	1.00	0.93	0.85	1.02	0.93/0.94	0.72	0.93	1.11	0.55	1.21	1.32	0.82	1.12	0.96
Leu		1.00		0.81	0.94	1.00	0.94/0.93		0.95	1.15	0.64			0.85	0.95	0.88
Lys		1.04	1.07	1.02	0.99	1.03	0.91/0.86	1.19	0.92	0.98	0.62		0.94		0.84	
Met		1.01	1.04	0.87	0.93	0.99	0.88/0.87	1.08	1.05	2.02	0.67				1.36	
Phe		1.09	1.08	0.83	0.89	0.98	0.92/0.90	1.00	1.00	1.21	0.78		1.30		1.04	
Pro		1.07	0.85		0.97	1.12	0.85/0.87	0.95	0.51	1.19	0.59				1.17	
Ser		1.01	1.13	1.11	1.10	1.04	0.92/0.89	0.86	0.98	1.14	0.79	2.10			0.87	0.93
Thr	1.48	1.01	0.94	0.92	1.07	1.08	0.89/0.94		2.46	1.00	0.63		1.08		0.93	0.82
Trp				0.82	0.93	0.94	0.94/0.88			1.02	0.61		1.15		1.02	
Tyr		0.98	1.09	0.89	0.92	0.96	0.92/0.80	1.55	1.07	1.20	0.56				1.24	0.78
Val		0.93	1.02	0.79	1.01	1.01	0.95/0.94	0.90	0.93	1.15	0.68	1.36		0.85	0.96	0.79
Cit			1.23	0.97	0.90	1.01					0.47				1.04	
Orn	1.28	1.07	1.47	0.65	1.25	1.12		0.98			0.53					0.68

Note: *—ER + PR+/TNBC.

**Table 3:** Metabolites associated with breast cancer risk.

No.	Author, Year	BC/Control	Subgroup	AAs	HR (95% CI)
1	Nagata C. et al., 2014 [88]	350	premenopausal	Arg, Leu, Tyr, Asp	-
2	Lécuyer L. et al., 2018 [89]	206/396	-	Val ↑	1.83 (1.15–2.92)
				Gln ↑	1.61 (1.02–2.55)
				Lys + Creatine + Creatinine ↑	1.84 (1.19–2.85)
				Lys + Arg ↑	1.62 (1.05–2.48)
3	His M. et al., 2019 [90]	1624/1624	-	Arg ↑	0.89 (0.80–0.99)
				Asp ↑	0.87 (0.80–0.95)
				Gln ↑	0.91 (0.84–0.99)
				Gly ↑	0.90 (0.83–0.97)
				His ↑	0.91 (0.84–0.99)
				Lys ↑	0.90 (0.83–0.98)
				Thr ↑	0.92 (0.85–0.99)
4	Zhang J. et al., 2020 [91]	735/735	-	Orn ↑	0.70 (0.53, 0.94)
5	Zeleznik O.A. et al., 2021 [87]	1997/1997	premenopausal	Ile ↑	0.86 (0.65–1.13)
			postmenopausal	Ile ↑	1.63 (1.12–2.39)
6	Jobard E. et al., 2021 [92]	791/791	premenopausal	His ↑	1.70 (1.19–2.41)
				Orn ↑	1.43 (1.06–1.95)
				Leu ↑	1.37 (1.01–1.86)
				Gln ↑	1.33 (1.00–1.78)
				Glu ↑	1.34 (1.00–1.79)
7	Stevens V.L. et al., 2023 [93]	1687/1983	-	Ser ↓	0.89 (0.83–0.96)
				Asp ↓	0.91 (0.84–0.97)
				Gln ↓	0.91 (0.85–0.98)

Note: ↑—amino acid concentration in breast cancer increases; ↓—concentration decreases.

## Data Availability

Not applicable.
